# Co‐Designing and Evaluating a Digital Competencies Toolkit for Nursing Students

**DOI:** 10.1111/jan.70329

**Published:** 2025-10-28

**Authors:** Konstantina Martzoukou, Errol Sadullah Luders, Moisés Piedade Henriques, Khairun Nisah Kamaruzaman

**Affiliations:** ^1^ School of Law and Social Sciences Robert Gordon University Aberdeen UK; ^2^ School of Health Robert Gordon University Aberdeen UK

**Keywords:** curriculum planning attitudes, evidence‐based practice, nurse education, nursing students, professional development, skills mix

## Abstract

**Aim:**

To offer a student‐focused critical evaluation of the content and use of a digital competencies discipline‐specific toolkit that was co‐designed with students, offering ideas for training and development across several digital skills areas, such as digital creation, research, communication, innovation, and wellbeing.

**Design:**

A cross‐sectional empirical study.

**Methods:**

The toolkit was evaluated based on clarity, level of comprehension, accessibility, perceived relevance, and future implementation through a survey, which collected quantitative and qualitative data from 339 undergraduate nursing students in a single school and university in Scotland. Original research data were collected in June 2023.

**Results:**

Students evaluated the toolkit positively for its clarity, comprehensive nature, and practical resources, but suggested improvements for neurodivergent learners. Most students recommended implementing the toolkit early in their course and emphasised its benefits in continuous use. The toolkit was found to be relevant for practice placements and career development. Despite study workload concerns, students were positive about upskilling, highlighting the utility of the toolkit.

**Conclusion:**

Digital literacy is essential as healthcare increasingly relies on digital tools, behaviours and processes. This study employed co‐design strategies, supporting students to act as co‐producers, change agents, and partners in learning.

**Implications for the Profession and/or Patient Care:**

The study highlights the need for continuous education in digital skills with suggestions for incorporating advanced skills for future practice, such as data analytics and artificial intelligence, and discusses the value of digital skills development in higher education to enhance student learning and future practice.

**Impact:**

The research offers insights of international relevance into the development of a digital competencies toolkit that proposes nursing‐specific educational digital skills interventions. The work fosters inclusivity, continuous digital skills improvement, and professional readiness.

**Reporting Method:**

The work followed the Equator Standards for Quality Improvement Reporting Excellence in Education.

**Patient or Public Contribution:**

No patient or public contribution.

**Summary**

- What already is known
  ○ Research with nursing students reports low competency levels in digital skills.
  ○ There is a need for targeted continuous professional development training programs to empower nursing educational curricula.
- What this paper adds
  ○ Proposes a student‐focused co‐creation approach to supporting digital skills development.
  ○ Empowers students to act as co‐producers, change agents and partners in learning.
  ○ Amplifies the student voice and suggests support for underrepresented students.
- Implications for practice/policy
  ○ There is a need for nursing specific digital literacy interventions in curricula.
  ○ Co‐design of digital skills resources with students can empower nursing education.
  ○ Collaboration between educational institutions can support the development of more impactful digital skills provision.
- Impact statement
  ○ This research supports nursing education by introducing a co‐designed digital competencies toolkit that enhances student professional preparedness for digitally driven healthcare environments.
  ○ The toolkit fosters critical thinking around digital tools, skills development, and digital behaviour, encouraging students to evaluate, apply, and innovate with digital resources in clinical and academic contexts. It promotes inclusive, student‐led learning and continuous digital upskilling, empowering students to become confident, reflective, and active partners in their digital learning and future careers.
  ○ The toolkit offers actionable insights for integrating student‐led digital literacy interventions into nursing programs, with potential for broader application across health education disciplines.

## 1 Introduction

The National Health Service (NHS) in Scotland has identified when, why, and how to use digital technologies as an area of priority for the health care sector (Scottish Government 2021a). However, a large‐scale digital skills research study by NHS Education has highlighted a lower‐than‐expected perceived value of time spent to develop digital literacy skills, coupled with a need for formal training (Capgemini 2022, 28–30). Wider research at the European level has revealed that, in relation to the development of digital skills, there is still ‘limited reference to the health professions’ and a need to both develop targeted training programs and empower educational curricula (Morrison and Rooney 2017). Other research has found that healthcare students have basic IT skills and self‐report low competency levels in digital skills (Brown et al. 2020; Lam et al. 2016; Martzoukou et al. 2023). The lack of digital literacy creates significant challenges for nursing students (Saeed and Masters 2021), even at the baseline level (Lokmic‐Tomkins et al. 2021). The assumption that digital natives possess high digital literacy has been proved wrong (Reid et al. 2023).

Digital literacy education is necessary to deal with an increasingly digitised education and the fast development of technology in the professional sphere, which requires everyone involved in the health system to be digitally literate (Kennedy and Yaldren 2017). The Topol Review (2019) highlights several recommendations for healthcare and the need for a more technological and digitally centralised curriculum. The regulating and awarding body for healthcare professional standards in the U.K. includes ‘digital and technological skills’ (p. 12) in the core standards of proficiency for registered nurses, for handling ‘digital information and data’ (p. 28), ‘effectively and responsibly’ using ‘a range of digital technologies’ (p. 20) and ensuring ‘safe and effective nursing practice’ (Nursing and Midwifery Council 2018, 9). A focus on critical digital themes in healthcare students' curricula could benefit the future healthcare workforce (Matthews 2021) and enhance patient care (Kennedy and Yaldren 2017; Pearce 2017).

## 2 Background

### 2.1 The Problem

Several studies, conducted in diverse countries, such as Turkey, the UK, Norway, South Africa, and Australia, address diverse digital literacy gaps in nursing students, demonstrating that this is a global concern, given the increasing integration of technology in healthcare (Erdat et al. 2023; Matthews 2021; Nes et al. 2021). For example, Nes et al. (2021) notes a lack of focus on transferable digital skills in Norwegian students, such as problem‐solving and critical thinking skills, while research in Turkey points to the variations in existing measurement scales and digital skills addressed (Erdat et al. 2023). There appears to also be an observed gap in advanced or specialised digital skills development in studies addressing South African and Australian students (Harerimana et al. 2022; Brown et al. 2020).

In relation to digital literacy frameworks used for mapping essential digital skills in nursing for developing training, a comprehensive review of previous studies across several high‐income countries (e.g., Canada, Australia, U.K., Singapore, Taiwan) reveals that there is a lack of emphasis on digital soft skills for working efficiently (e.g., virtual collaboration) and driving creative solutions through technology. A scoping review by Nazeha et al. (2020) identified a total of 30 health‐related frameworks, most of which addressed the domain of informatics (e.g., basic computer knowledge, information systems, and the ability to maximise the use of digital technologies), while only one framework addressed telehealth. More emphasis could be placed on digital communications with patients (e.g., virtual consultations, email) as well as other behavioural aspects, such as digital collaboration, productivity, and innovation. Overall, Nazeha et al. (2020) called for digital health frameworks to ‘be regularly updated with novel digital health technologies’ (such as health apps, artificial intelligence, autonomous decision‐support systems) and to address more holistically all the requirements of the Health and Care Digital Capabilities Framework (Royal College of Nursing 2017).

### 2.2 Further Development Needed

There is still a need for more tailored and discipline‐specific educational resources that address diverse digital skills to better prepare students for the digital demands of modern healthcare environments. Research shows that nursing students report low competencies in critical areas, such as information literacy, digital research, and digital innovation, while there is a need for targeted educational digital skills training interventions to prepare them for comprehensive patient care (Martzoukou et al. 2023, 2024).

Previous research mainly consists of explorations around current digital and eHealth literacy gaps in students, using multifaceted assessment tools (Karnoe et al. 2018) that examine students' familiarity, use of technology, and ability to actively engage with digital services (Holt et al. 2020), or the relationship between digital capabilities and academic performance, self‐efficacy, and success (Ibrahim and Aldawsari 2023). However, there have only been few attempts to develop digital literacy toolkits with resources that are tailored to the specific learning needs of nursing students and that engage them directly in the co‐creation and evaluation of learning material.

Our approach, discussed in this paper, consisted of developing a toolkit with digital literacy resources that was informed by existing digital literacy gaps identified by nursing students, and invited learners' perspectives, supporting an inclusive approach to engaging with the student voice (Bovill 2013). Central to the toolkit development was a tailored, discipline‐related, and student‐based approach, working closely with students to engage them directly in its development. Co‐creation strategies in higher education (such as co‐planning/designing learning activities and co‐producing knowledge) can create mutual value for both students and universities as students can act as co‐producers, change agents, and partners in learning (Zarandi et al. 2022). A co‐designed curriculum can also lead to transformational experiences (e.g., positive relationships and community engagement; overcoming challenges) (Lubicz‐Nawrocka and Bovill 2023). In addition, the toolkit addressed not just a local, but a universal need for providing a structured and discipline‐based approach to developing essential digital literacy skills, bridging digital literacy divides in nursing students. It fostered the adoption of digital health technologies (e.g., telehealth) and behaviours (e.g., creativity, innovation, and continuous learning), which are crucial for modern healthcare systems. The ability to effectively use new digital tools and develop technology‐related responsible and efficient behaviour for enhancing patient care is crucial for nursing professionals worldwide.

Although the work presented in this paper was designed within a specific education context and student cohort, its structure and content can be adapted for different educational settings and cultural contexts, which makes it a valuable resource for nursing education programs internationally.

### 2.3 Existing Digital Skills Development Solutions

Different academic institutions and public organisations have implemented diverse projects for identifying nursing students' digital skills gaps within an increasingly complex digitised healthcare landscape. Insightful overviews by Matthews (2021) and Nes et al. (2021), showcase a variability of approaches and directions, including computer literacy, health/nursing informatics, and technology acceptance. Of particular interest to this study, however, is the development of practical toolkits that offer a ‘knowledge translation strategy used to communicate messages or share decision aids, tools, or resources to improve health, educate, or change practice/behaviour’ (Godinho et al. 2021). Toolkits have a practical nature as a means of providing access to resources, learning activities, programmes, training, and support that can enhance understanding, skills, and competencies. A toolkit, as approached in this study, is, therefore, not just a theoretical framework or a roadmap of digital skills, but a means to develop skills with adaptable resources and training interventions. Toolkits can be customised by a specific context, area, or discipline, helping to bridge the gap between theory and practice, improving both student academic outcomes and employability.

A brief review of the literature on digital skills toolkits demonstrates that they examine a range of skills from telemedicine and remote health consultations to digital professionalism, the use of technologies, handling patient health data, and e‐prescribing. However, while existing digital literacy toolkits include learning resources for healthcare education, addressing a diversity of digital literacy perspectives from using technology to eHealth Literacy (HL) (Patil et al. 2021; Health Education England 2016), they lack a focus on nursing students. An exception to that is the work developed by The Canadian Association of Schools of Nursing 2012 and Canada Health Infoway (2012), which includes e‐resources for both students and nursing faculty on ICT skills, patient care delivery, information management, professional accountability, and the use of technologies in clinical practice, emphasising the intersection of informatics and nursing care. Another nursing‐specific approach is in the form of LibGuides (Stankus and Parker 2012), developed by academic libraries. For example, the ‘Nursing Tools’ LibGuide by Duke University Medical Centre Library and Archives (2024), provides resources for evidence‐based practice and clinical nursing digital tools, eBooks, and freely available mobile apps for clinical practice. However, LibGuides mainly focus on digital research skills and are underused by students (Carey et al. 2020).

Higher Education institutional projects may also address professional digital skills development for students, but, again, these are typically aimed at broader groups, such as medical/health education students. For example, the TEPE (Technology‐Enabled Practice Education) Project, developed by Trinity College Dublin (Lynch et al. 2023) focused on the development of clinical skills for technology‐enabled practice, including interactive learning resources in diverse areas, such as digital professionalism, digital literacy, data protection and management (Lynch et al. 2022) with a broad focus on students within health and social care. Digital professionalism, ‘the competencies and values expected of professionals when communicating online’ (Smart et al. 2022) has also been explored by (Chretien et al. 2024), aimed at medical professionals with the support of the Association of American Medical Colleges (AAMC). The project developed case studies focusing on difficult online professional situations, a case commentary on handling social media issues (e.g., anonymity, accountability) and offered questions for critical discussion.

In the U.K., the Health Information Literacy Toolkit, developed by NHS England (2023a), has, similarly, focused on the digital health literacy of healthcare professionals as well as patients. It combines digital health literacy skills, critical appraisal, and social/communication skills for promoting health, providing access to techniques, e‐learning courses, guides, and guidelines focusing on consumer health. The Digital Skills Assessment Tool (DSAT), developed by NHS England (2022), has been another project aiming to improve the digital literacy of the health and social care workforce. It addresses self‐assessed ‘digital readiness’ (p. 4) for information, data, and content, teaching, learning and self‐development, communication, collaboration and participation, technical proficiency, creation, innovation and research, digital identity, wellbeing, and safety and security. Other work includes the Digital Champions toolkit, which is aimed at social care leaders to promote digital literacy and support other colleagues. It includes different case studies on the use of apps and digital technologies that have been implemented across geographical locations in the UK by different councils and organisations (NHS England 2022). The ‘Digital Capabilities for the Pharmacy Workforce’ (NHS Health Education England 2021) is another toolkit that provides advice, resources, and courses on clinical informatics and remote consultation skills in healthcare settings.

The above discussion provides evidence of an existing gap in the availability of digital literacy toolkits aimed at nursing students. A systematic detailed overview of research on digital competences in Higher Education by Matthews (2021) points to a need to address digital literacy development as ‘a curriculum component’ (p. 8) in Higher Education. Furthermore, there are areas of digital literacy in the guidance by Health Education England (HEE) and the Royal College of Nursing (RCN) that are not addressed in curricula, pointing to a need for a more holistic integration of digital skills as core learning outcomes. Digital literacy programmes should also be personalised to accommodate the diverse skill levels of students entering Higher Education.

## 3 The Study

### 3.1 Aim

The aim of this study was to critically evaluate a Digital Competencies Toolkit (DCT) that was developed for the purpose of supporting nursing students' digital literacy skills development. The toolkit was designed based on previous empirical research with nursing students, which identified self‐assessed digital competencies gaps for nursing‐related learning and future practice.

### 3.2 Objective

The evaluation study focused on the following objective: To critically evaluate the design, content, and use of the DCT with nursing students, exploring the potential impact of this work on students' learning and future practice.

We addressed the following research questions:

- What key features and resources should be included in a digital competence toolkit for nursing students?
- How can the toolkit be designed to be user‐friendly and accessible for nursing students with varying levels of digital literacy?
- What are the best practices for integrating the digital competence toolkit into existing nursing curricula?
- What strategies can be implemented to ensure the long‐term sustainability and relevance of the toolkit?

## 4 Methods

### 4.1 Context

The study was conducted in the context of a Scottish university that maintains a strong emphasis on employability, high‐quality teaching, cross‐disciplinary research, and promoting student‐staff partnerships. The research reflects the specific educational environment of nursing students within that university, geared towards fostering innovation, interdisciplinary collaboration, and practice‐based learning. In addition, the research aligns with the priorities of the Scottish Government's Digital Strategy on integrating digital literacy into the curriculum as a core competence for all learners (Scottish Government 2021b) and its ‘Digital Health and Care Strategy’, placing emphasis on recognising digital skills as core competencies for healthcare professionals (Scottish Government 2021c). These, however, are priorities that are shared globally and not necessarily only confined to a Scottish context.

### 4.2 Intervention

#### 4.2.1 Co‐Development of the Intervention Content

The intervention was based on the outcomes of previous empirical research that was conducted by the research team in the same setting to explore nursing students' digital competencies gaps, using a self‐assessment Digital Competencies Survey (DCS). Despite the local character of the setting, the DCS was developed based on the European Digital Competence Framework for Citizens (Carretero et al. 2017) and the Digital Capabilities Framework (JISC 2022) that were adapted to holistically explore digital competencies from a nursing‐based perspective. The DCS explored competencies that relate to students' learning, future work, and everyday life. The structure and the dimensions of the DCS are presented in Table 1.

**TABLE 1:** Structure and dimensions of the Digital Competencies Survey (Martzoukou et al. 2024).

Everyday participation as a digital citizen (Items *N* = 9)	E‐democracy (e.g., accessing voting information and political information online; taking an active role in democratic processes online) E‐government (e.g., obtaining knowledge about current laws, legislation and government, accessing and using government online services, such as legal information) E‐finance (e.g., online banking, price comparison websites, managing personal/student finance); e‐commerce (e.g., online shopping, buying & swap apps) E‐commerce (e.g., online shopping, buying & swap apps) E‐health (e.g., accessing and using health services online, e‐consult with doctors, NHS 24 online services) E‐wellbeing (e.g., personal health tracking, e‐fitness, e‐mental health self‐management) E‐leisure (e.g., playing online games, socialising online) E‐learning (e.g., looking for new digital opportunities to grow as a person such as online webinars, online training, watching YouTube videos and following an active approach to sourcing information) E‐employment (e.g., working remotely, using digital content and tools for work purposes)
ICT proficiency with completing different tasks (Items *N* = 6)	Technological devices (e.g., laptops, tablets, smartphones, desktop computers; connecting to the Internet/wi‐fi) Web browsers (e.g., Chrome, Explorer, Firefox, Safari etc.) Search engines (e.g., Google, Bing etc.); University digital administrative services (e.g., email, student data portal) University digital administrative services (e.g., email, student data portal) University learning management systems (e.g., Moodle, Blackboard, Brightspace) Communication Platforms (e.g., Zoom, Skype, Microsoft Teams, Google Hangouts)
ICT productivity (items *N* = 5)	Organising/managing/storing your digital files effectively for your learning (e.g., using filenames and through folders) Sharing securely your digital files with others (e.g., sharing files on Moodle, via email) Using productivity tools, such as calendars, task lists, project and time management apps, to make learning more efficient (e.g., Microsoft Project, Outlook/Google calendar, Trello, Toggl) Proof reading/spell‐checking your work Creating formatting styles (e.g., Table of Contents, report‐writing styles)
Information literacy (identification of information types) (items *N* = 3)	Scholarly/academic literature (e.g., journal articles, conference papers, book chapters, other publications written and vetted by subject experts) Professional literature (e.g., Professional organisations such as Nursing and Midwifery Council publications, Royal College of Nursing Publications, Royal College of Midwives Publications, Health Professional Blogs, The Royal College of Paramedics, Scottish Government publications/policy) Popular information (e.g., general discussions on social media, websites and blogs)
Information literacy skills (items *N* = 9)	Finding digital information relevant to your academic studies, using informal Web sources (e.g., Google, Google Scholar, Bing or other search engines) Finding digital information relevant to your academic studies, using databases (e.g., CINAHL, Medline, Science Direct, Cochrane Library) Using online collection tools for gathering digital information together in new ways (e.g., Slideshare, List.ly, Pinterest, Quora, Scoop.it, etc.) Evaluating whether digital information is trustworthy and relevant; organising the digital information you find for your learning through folders, bookmarks, reference management software, and tagging Understanding academic integrity/honesty when accessing & using information online (e.g., plagiarism, collusion) Understanding how to share information publicly online, respecting and acknowledging the work of others (e.g., using creative commons licensing, providing references/citations to original works) Using artificial intelligence generated content ethically, following academic integrity values (e.g., using ChatGPT, Google Bard) Referencing digital information sources, adhering to a referencing style (e.g., Harvard referencing style)
Digital creation skills (items *N* = 8)	Creation and editing of videos Infographics (e.g., Canva) Online posters Blogs/Wikis Vlogs/Podcasts Creation of audio files (e.g., using Audacity, Voice‐over presentations) Using Simulation/Virtual Reality Tools (e.g., virtual hospital/community) Data visualisation (e.g., Excel, SPSS)
Digital research skills (items *N* = 8)	Finding research raw/open data online (e.g., open health data, national statistics sources such as the Scottish Public Health Observatory, Information Services Division Scotland, The World Health Statistics) Organising and storing research raw/open data online (e.g., using tools such as RefWorks or Microsoft Word to annotate or summarise findings) Using a Critical Appraisal Tool (e.g., CASP) Using a survey tool (e.g., Online Surveys, Mentimeter) Analysing digital research data using simple tools (e.g., spreadsheets, textual data analysis software, visual tools) Using methodologies to cleaning, transforming and preparing open data sets (e.g., available on the Internet, via different organisations, research institutions) Understanding how evidence‐based research are used to construct arguments, make decisions, and/or solve problems Following ethical, legal, and security guidelines when using research data (e.g., Social, Ethical and Professional Guidelines, personal data protection regulations such as GDPR)
Digital communication skills (items *N* = 9)	Participating professionally (e.g., reviews, comments, likes) in a range of digital networks (e.g., social and professional networks) related to your interests, work, and/or academic subject Understanding expected behaviour/code of practice in online environments (e.g., NMC/HCPC Social Media guidance) Communicating respectfully, inclusively & confidentially, recognising that digital media can be used to intimidate, shame, and harass other people Communicating professionally via email with others (e.g., peers, tutors, mentors) Actively participating in online learning environments (e.g., discussion forums) Recognising false or damaging online communications (e.g., fake news, misinformation) Actively sharing your specialist ideas (e.g., academic or professional) in a range of online communication media (e.g., social media such as LinkedIn, Twitter, Facebook) Sharing information using external communication tools (e.g., WhatsApp, Viber, Skype) Designing online communications for different purposes (e.g., online discussions, blog messages, X (Twitter) threads to persuade, inform, entertain, guide, and support)
Digital innovation (items *N* = 4)	Developing new ideas and projects using online tools and technologies (e.g., using tools in innovative ways to create presentations, projects, apps, video resources and designs) Engaging with professional digital innovations (e.g., telehealth initiatives, the use of smartphones and health online applications for consultations and patient care) Working collaboratively on different aspects of a creative/innovative project/service design & managing the process as a team Promoting new online tools and opportunities to others (e.g., proactively promoting creative ideas and projects)
Digital learning and development (items *N* = 8)	Participating in online learning opportunities and resources (e.g., online courses, podcasts, global conversations on X (Twitter), quizzes, online tutorials, simulations, or open lectures) Adopting new ways of learning online (e.g., online workshops, virtual labs, video‐tutorials, webinars) Working collaboratively and supportively with other learners, using online technologies where appropriate (e.g., via your university's online education system (Moodle), Office 365, other apps and online environments or via your previous working experiences) Using online tools to take notes, annotate, and collate learning materials, review, and revise learning (e.g., Evernote, Notion, Google Apps, Scribble) Using online tools to record learning events/outcomes and use them for self‐analysis, reflection, and showcasing of achievement (e.g., in an e‐portfolio or learning blogs) Receiving and responding to online feedback about your academic work Using learning management systems (e.g., BlackBoard Collaborate, Zoom, Teams) to learn collaboratively Sharing your online knowledge and skills, helping other learners (e.g., mentoring others)
Digital identity management (items *N* = 7)	Managing your online profiles on different digital media (e.g., social media) in a way that is suitable for personal, professional, and academic purposes Understanding how your online personal data are collected and used in different systems and use privacy settings appropriately Being aware of the potential positive or negative impact of what you communicate online on your online reputation Making sure outcomes of learning and other achievements are accessible in online forms (e.g., via an e‐portfolio, digital CV, personal website) Understanding the impact of your online interactions (e.g., how you project yourself to others online) Using online analytics to explore your impact and influence on others Establishing healthy boundaries/habits in using social media (e.g., monitoring time spent online).
Digital wellbeing (items *N* = 6)	Feeling comfortable, in control, and safe when using digital technologies Recognising that digital information and media can cause distraction, overload, and stress, and disconnecting when necessary Considering the rights and wrongs and the possible consequences of your online behaviour Acting positively against cyberbullying and other damaging online behaviours Managing online and real‐world interactions in ways that support healthy relationships Using digital media to access wellbeing services, monitor health conditions (e.g., student support services)
Digital abilities to complete academic work (items *N* = 1)	Which level best describes your digital abilities to complete your academic work (e.g., using digital tools and processes as set in your course)?

The DCS uses a 5‐point Likert scale, from Level 1 Novice: ‘The digital task is new to me. I am currently developing basic knowledge and skills in this area, but I need help either to complete or to learn how to complete this sort of task’ to Level 5: Expert: ‘I have mastered the knowledge and skills for this area. I apply my knowledge and skills to create and redesign processes, tools, and/or technologies appropriately and effectively. As an expert in this area, I frequently show others how to complete these tasks’ (Martzoukou et al. 2020).

Through two rounds of earlier empirical research using the DCS, nursing students reported low competencies in several digital literacy dimensions, including information literacy, digital research, and digital innovation, highlighting the importance of embedding digital literacy within nursing study programs to prepare them for comprehensive patient healthcare (Martzoukou et al. 2023, 2024). The empirical research also provided key recommendations for following tailored, discipline‐related, holistic, practice‐based, and curriculum‐embedded approaches to students' digital skills development. Based on these results, it was important to develop a process by which, once students self‐identified their individual digital competencies gaps, they could gain access to resources for training and development across the digital competencies areas of the DCS.

The Digital Competencies Toolkit (DCT) was, therefore, designed as an intervention to the outcomes of the DCS with the purpose of integrating it into the university's nursing curriculum, comprising various courses, such as adult, mental health, and children's nursing. The DCT was developed to support nursing students in upskilling in the digital competencies areas of their choice, offering ideas for training and development across several digital skills areas, such as digital creation, research, communication, innovation, and wellbeing.

#### 4.2.2 Delivery of the Intervention

**FIGURE 1:**
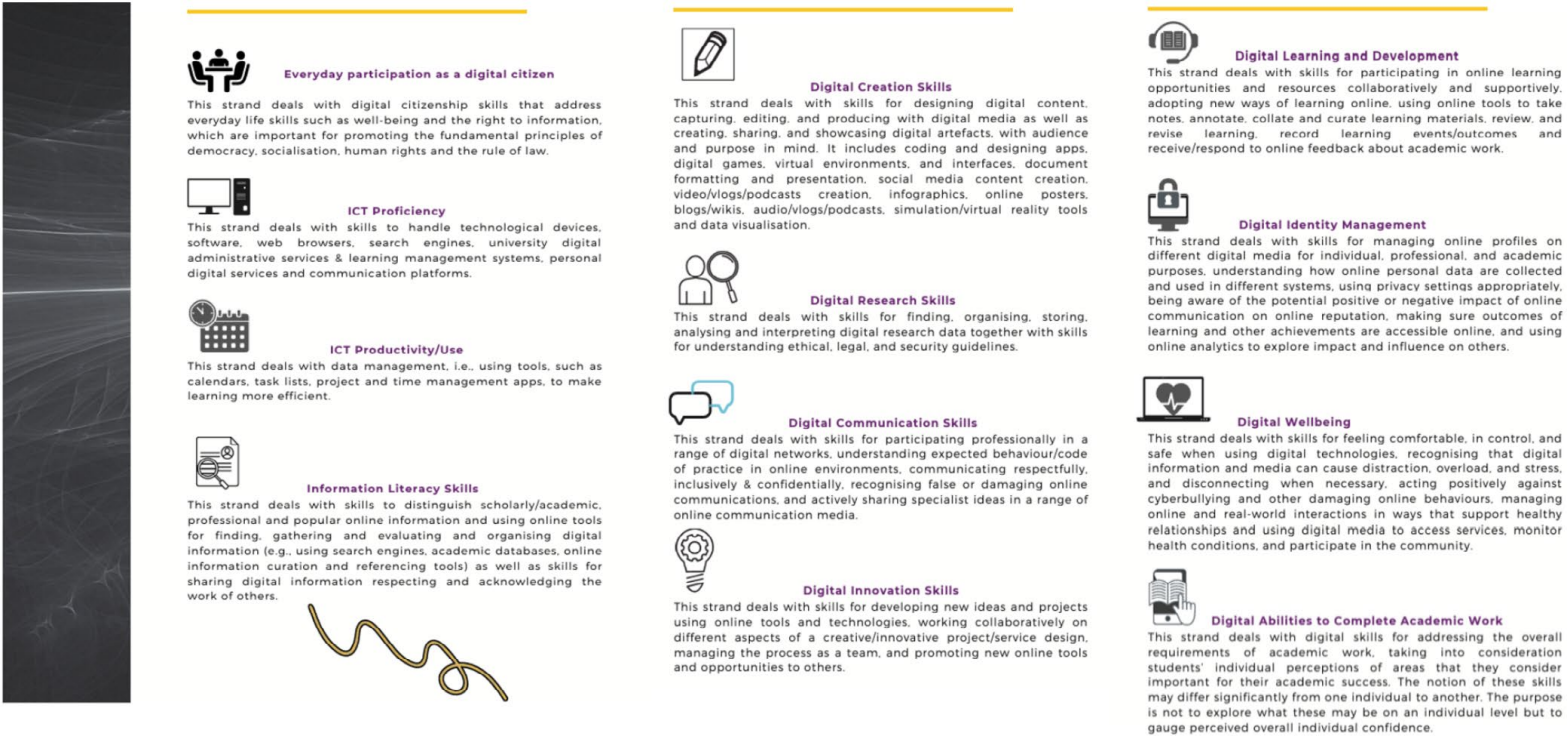
Digital competencies strands (DCT).

The DCT was created using the software Canva (2024), which is downloadable in the form of a PDF document, or it can be shared online. An e‐toolkit version was also made available via ‘Visual Paradigm’ (2024), a tool that converts PDF into an engaging e‐book/flipbook version that is easy to read and share. Figure 1 provides an overview of the DCT strands.

**FIGURE 2:**
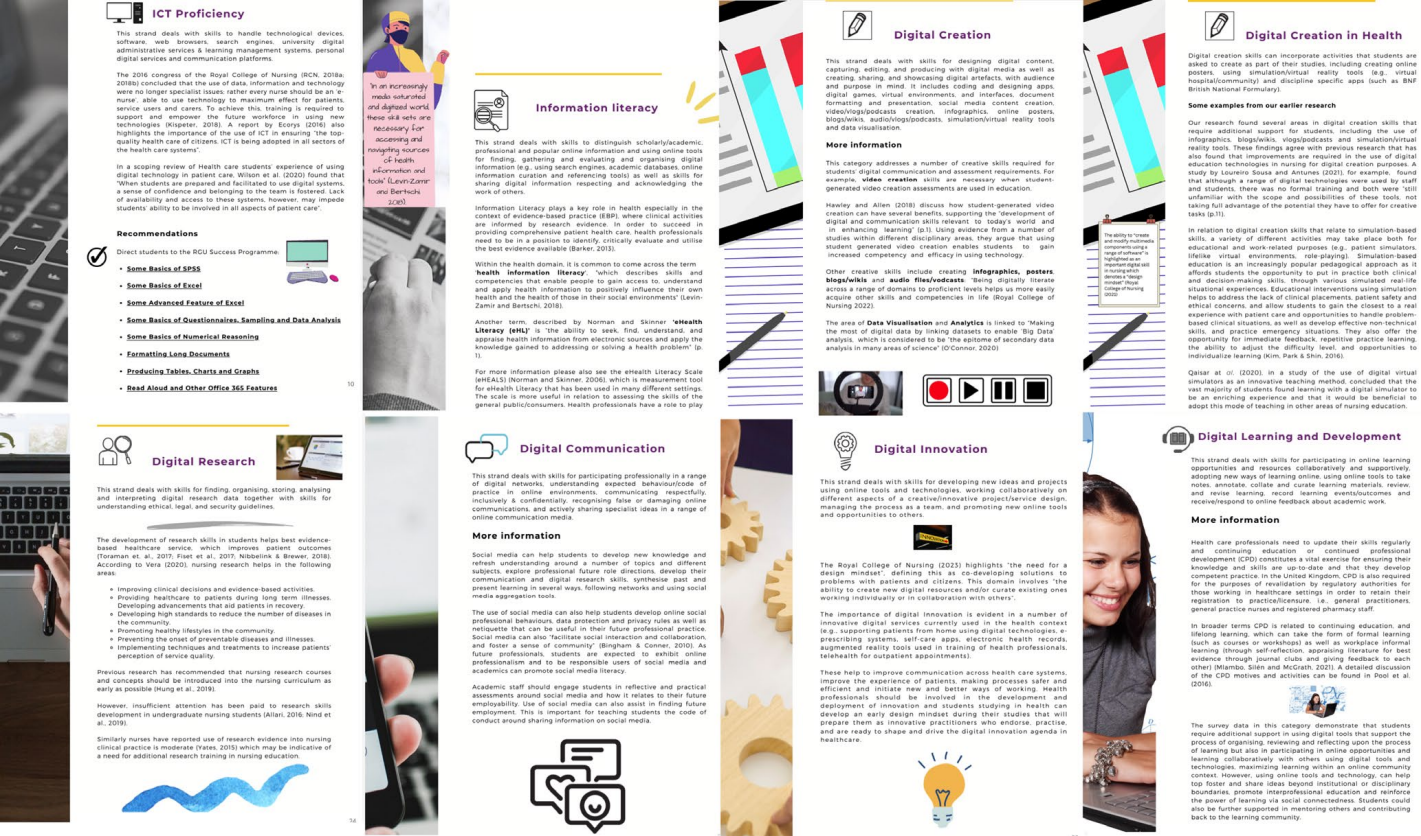
Indicative Examples of Introductory Pages in the DCT Toolkit.

Following each stand, students are provided with an explanation of why digital skills are important with a theoretical background, introducing the value of different digital skills for learning and nursing‐related practice (Figure 2).

**FIGURE 3:**
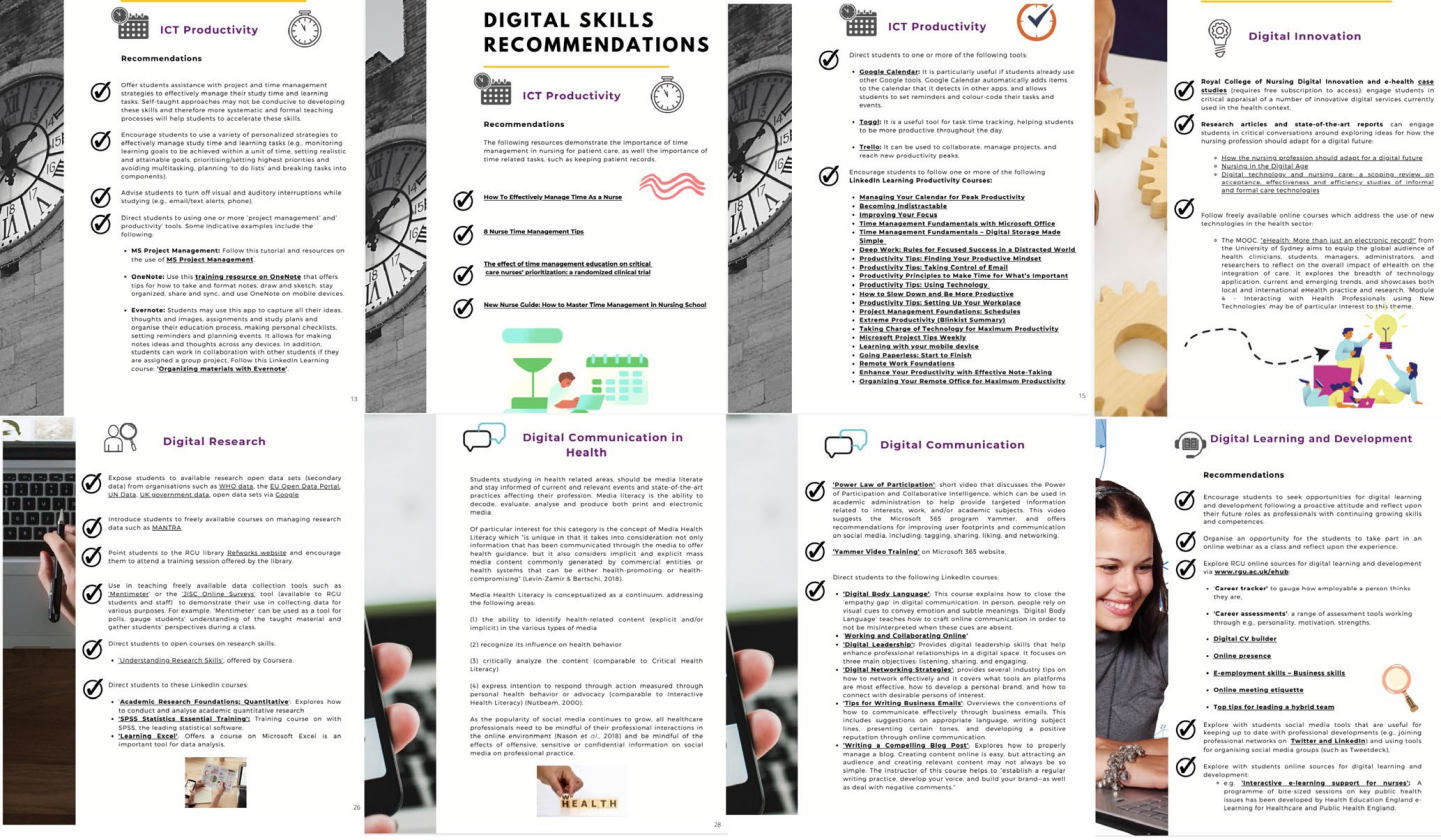
Indicative examples of recommendations in the DCT toolkit.

Following the introductory pages, students are offered a set of upskilling recommendations. For example, recommendations for ‘ICT productivity’ include advice for assisting students with project and time management approaches, using a variety of personalised strategies to effectively manage study time and learning tasks (e.g., monitoring learning goals to be achieved within a unit of time, setting realistic and attainable goals, prioritising/setting highest priorities and avoiding multitasking, planning ‘to do lists’ and breaking tasks into components), and a variety of training courses. Other recommendations highlight the importance of time management in nursing for patient care and time‐related tasks, such as keeping patient records. Recommendations for ‘Digital innovation’ encourage students to engage in critical conversations on how the nursing profession should adapt and think critically about the overall impact of e‐health, while recommendations for ‘Digital wellbeing’ address students' everyday life and academic strategies for keeping digitally healthy (Figure 3).

The DCT was developed with the direct input of four MSc Information and Library Studies masters' students, interested in future health librarian careers, who were recruited for the duration of one academic semester (which lasted 3 months) for the purpose of updating, collating, and evaluating resources for the toolkit, as part of a credit‐bearing module, ‘Professional Skills Enhancement’. The module is designed with the purpose of allowing students to contribute to a practical project (internal or external) and identify strengths and development areas for personal development. As part of the module, students attend bi‐monthly online meetings with their academic or industry supervisor, who is responsible for the project, and the students' work is assessed based on a professional reflective report, which offers a critical appraisal of practical skills and theoretical knowledge developed.

Highlighting the importance of interprofessional cooperation, library students were tasked with the following objectives: (a) to help with the updating of the resources in the online toolkit, checking the existing links and projects, (b) to tailor the toolkit to the area of nursing, adding descriptions, research, and projects to the toolkit related to that area, and (c) to update the work with a visual appeal for nursing. Students were able to develop an understanding of digital skills development in an area of nursing, advance their research skills, develop information retrieval, evaluation, and data curation skills, and learn how to edit work to a professional standard using digital tools.

The practice of working closely in partnership with students to co‐design educational material was supported by both the university strategic direction for cross‐disciplinary research and scholarship, as well as previous research on pedagogy which advocates that academic student partnership in the co‐creation of education material fosters an environment of deeper learning experience for students, a higher level of commitment and engagement with learning (Deeley and Bovill 2017; Doyle et al. 2019; Draper 2009). Our approach invited learners' perspectives, motivated active learning, and enhanced learners' feelings of engagement with the topics explored, as well as creating a sense of ownership and student empowerment. We also supported students' belonginess (Bovill 2013) with an inclusive approach to engaging with the student voice and empowering student agency. The participatory and collective learning design also helped students to identify digital skills areas of importance and created the foundations for fostering the importance of critical reflection and the values of developing into a reflective professional. For example, as one of the participant students reflected, in their reflective submitted report, the project allowed them ‘to understand the needs and perspectives of nursing students using the toolkit in the future’, putting themselves ‘in their shoes and question the relevance of the information for their specific setting’, ‘build confidence’ and develop ‘IT literacy’ and ‘active listening’ skills.

### 4.3 Study of the Intervention

The study was conducted in June 2023, in the form of a cross‐sectional survey design involving the collection of qualitative and quantitative data from nursing students to evaluate the content and use of the DCT. Total population sampling was followed to email survey invitations to 964 Undergraduate (UG) students and 32 Postgraduate (PG) registered students. The final sample consisted of 339 students in different years of study (Year 1, 2, and 3) and followed a variety of nursing courses.

The student‐based evaluation process explored the potential limitations and strengths of the toolkit as well as its future application in student‐based learning. The nature of the cross‐sectional survey, which captured data at a single point in time, can make it challenging to confidently generalise the findings to other student groups. However, bias was minimised by means of the broad representation of the sample, which included students in different study years and courses. Quantitative data provided measurable results, while qualitative insights offered deeper understanding of the perceptions and experiences of the students around the perceived impact of the DCT, supporting the triangulation of the data. These combined approaches offered reasonable confidence in the findings, as they enhanced reliability and strengthened the evidence supporting the effectiveness of the intervention. To further strengthen the approach in the future, the analysis could involve pre‐ and post‐assessments of students' digital skills, comparing skill levels before and after using the toolkit or comparisons between groups of students who have received the interventions and those who have not to observe whether similar improvements have occurred.

### 4.4 Measures

#### 4.4.1 Description of the Approach

The survey included a mix of closed and open‐ended questions to gather both quantitative and qualitative data. Quantitative data were collected via closed‐ended 5‐point Likert scale questions (from Strongly Agree to Strongly Disagree) addressing the clarity of the toolkit content, its appropriateness and comprehensiveness, accessibility, perceived relevance for supporting students in their studies, their practice placements, and their professional career development. Students were also asked if they would recommend it to other students. The toolkit performance metrics were designed with the purpose of identifying both success and further development areas, such as the potential engagement/disengagement level of students with the resources provided, the level of comprehensive coverage versus gaps in content, and whether the toolkit could be smoothly integrated into student learning or required major modifications. Via open‐ended questions, students were asked to also consider any additional areas of digital skills that had been omitted, to consider the most ideal time to utilise the toolkit, to comment on what a ‘perfect’ toolkit would look like, and to suggest further improvements and ideas for the implementation of the toolkit. The survey provided valuable data that helped to set the parameters for ongoing engagement with the student voice and preparing the path to improving the process of students' digital skills development, using a student‐focused methodology. It contributed to the efficiency of the toolkit, shaping it into a student‐friendly and relevant educational resource, directly addressing the needs of students and reducing the need for subsequent extensive revisions. It also provided a cost‐effective and discipline‐relevant approach to integrating digital skills training into the existing learning environment of students, eliminating the need to outsource digital skills assessments tools that are not tailored to nursing. General digital skills frameworks, such as the one developed by JISC (2022), ‘lack guidance as to the underpinning values of a specific sector’, while there is a need to ‘scaffold the engagement of nursing students as they access and engage with digital learning processes’. As part of that, exploring their ‘prior digital experiences’ and ‘feelings, knowledge and motivation about digital skills’ (p. 131) is fundamental (Waight and Holley 2020, 129–131).

#### 4.4.2 Methods for Assessing Completeness and Accuracy of Data

The instrument was reviewed by a nursing academic and a nursing online learning developer to ensure that each question aligned with the theoretical framework used (the DCS) and that all dimensions of the construct were included, that it accurately assessed the intended constructs, and that the questions were clear and relevant, following a logical flow and structure without any ambiguous or confusing items. The reliability of the survey instrument was tested using Cronbach's alpha. The results showed that all item groups in the questionnaire had a Cronbach's alpha index much higher than 0.7, with all the values above 0.9, which indicates excellent internal consistency among the Likert scale questions.

Quantitative data were analysed based on descriptive statistics using SPSS (v28) (IBM Corp 2022) to determine the levels of students' agreement regarding the usefulness and relevance of the toolkit. For the qualitative data analysis, open‐ended responses were analysed thematically to identify common themes, suggestions, and areas for improvement. The thematic analysis started with data familiarisation, followed by generating initial key codes and looking for patterns (Kiger and Varpio 2020). Emergent codes reflected students' perceptions of the relevance (or lack thereof) of the toolkit to learning, future career trajectories, and broader professional and social aspirations.

#### 4.4.3 Ethical Considerations

The research project was approved by the Research Ethics Committee of the School, which addressed the ethical procedures followed according to its ethics policy, addressing anonymity, confidentiality, informed consent, the right to withdraw, data handling, privacy, and potential risks; for example, disconnecting the purposes of the research from academic outcomes (SERP reference number: 23‐06). The survey was conducted with student voluntary and anonymous participation and informed online consent.

## 5 Results

### 5.1 Characteristics of the Sample

Total population sampling was followed to email survey invitations to 964 Undergraduate (UG) pre‐registration nursing students and 32 Postgraduate (PG) registered students. The final sample consisted of 339 students, representing a total response rate of 34%. Students attended diverse courses covering adult, mental health, and children and young people's nursing, representing different perspectives on digital competencies development and experiences.

### 5.2 Digital Competencies Areas Selected

#### 5.2.1 Quantitative Findings

**FIGURE 4:**
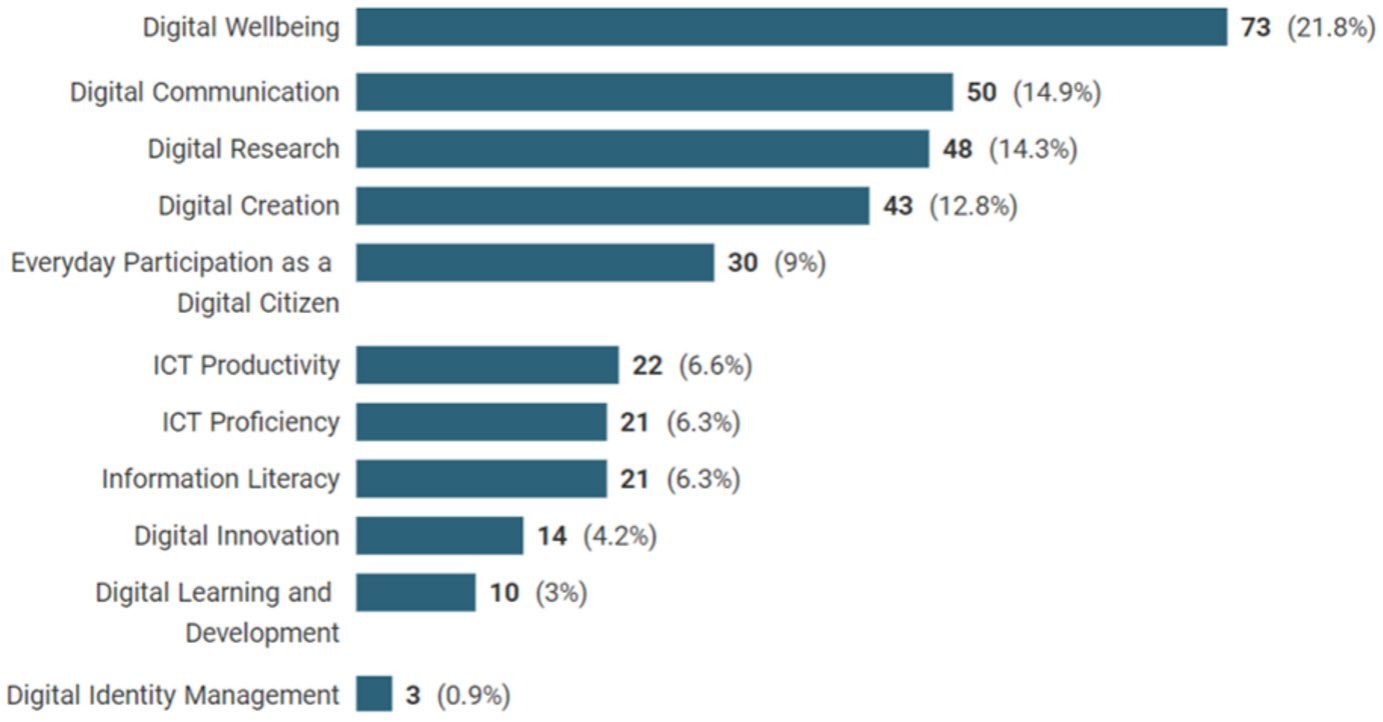
Digital competencies areas selected to be reviewed by students.

Due to the extensive length of the Digital Competency Toolkit (DCT), comprising almost 50 pages, it was not feasible for students to engage with the entire document. Consequently, each student was invited to select a specific digital competency area for focused review. The selection patterns offered insight into the domains of digital literacy that students perceived as most relevant or engaging. The most frequently chosen digital competence reviewed by students was ‘Digital wellbeing’ (*n* = 73, 21.8%), followed by ‘Digital communication’ (*n* = 50, 14.9%), ‘Digital research’ (*n* = 48, 14.3%), and ‘Digital creation’ (*n* = 43, 12.8%) (Figure 4). However, several students indicated that they found all the areas listed in the toolkit as valuable: ‘I can get a better understanding of how I can develop and become better with my digital skills’, ‘they all have an important part in digital skills’ and ‘I am not very confident when it comes to IT so I would recommend all areas’ (Figure 4).

#### 5.2.2 Qualitative Findings

Through open comments, students shared different perspectives on how the toolkit would help them increase awareness and broaden their understanding of diverse digital skills required in nursing: ‘I would for some of the competencies be interested in attending, as some I am very poor in and I'd love to develop my knowledge and skills’ and ‘I think it was useful to see and loot at and broadened my understanding’. Other students commented: ‘I would definitely use the toolkit to help improve my digital skills’ and ‘Would be useful for studying at university’ as it ‘Expands people's mind to education’ with ‘Good links to gain new skills’.

Additional comments described the toolkit as ‘a great piece of work’, ‘a very helpful and a great tool for students to have’. As students put it, ‘It already looks good for me and meets my needs as a digital information reader and learner’, ‘helpful and have downloaded a copied for future referencing’ and ‘I think it has all been covered well’. Other comments indicated that the toolkit covered ‘a wide range’ of skills and ‘I don't think I have anything I would change’. Indicatively, students further expressed that ‘I cannot think of any additional areas of digital skills to be added, the toolkit is very informative and useful and would really benefit students’, that ‘The toolkit is really easy to follow and very clear, it will be very beneficial to students’ and that ‘The toolkit covers all basic digital competencies in an easy to understand format—I think any more would be too much and would reduce engagement from students’.

Students' comments also highlighted several different areas where the toolkit would help them to recognise or reinforce specific digital skills, particularly in the areas of ‘Digital communication’ and ‘Digital wellbeing’, ‘Digital creation’, ‘Digital research’, ‘Information literacy’, ‘ICT proficiency’ and ‘ICT productivity’. Respondents referred to resources in the toolkit that addressed the use of Microsoft Teams for communication, the significance of ‘digital body language’ and ‘how to find your voice’ and the need for confidentiality in social media, as ‘communication isn't something I engage in much and appreciate the guidance. Also ties in with NMC [Nursing and Midwifery Council] values’. ‘Digital wellbeing’ resources highlighted included cyber security and physical wellbeing tips, such as ‘How to unwind and unplug ‘and ‘deal with digital distractions’, while when reviewing ‘Digital research’ resources, students emphasised practical skills and reliable sources, ‘Reinforcing the practical skills of digital research’ with ‘more practical sessions on how to navigate digital research.’ Information literacy resources helped students ‘with essay work to ensure academic sources are valid’ and with referencing. ‘Digital creation’ resources were deemed necessary for making presentations and videos, while ‘ICT proficiency’ was deemed important in advancing Excel skills and project management, developing essential IT skills for everyday life and workplace success (Table 2). On the other hand, ‘Digital innovation’, ‘Digital learning and development’ and ‘Digital identity management’ did not appear to be equally areas of interest.

**TABLE 2:** Digital competencies toolkit: areas students would use.

Digital communication	‘I would use it as digital communication isn't something I engage in much and appreciate the guidance. Also ties in with NMC values’.	‘I will use the core features of Microsoft Teams and how Teams are brought together to create conversations and content’.	‘Using Microsoft teams to communicate with peers and lecturers. I prefer learning in person, but this way is simple to understand and easy to use’.	‘I thought the digital communication section is something I can go back to as it identifies actual [university] sources I can use’	‘Working in an online study group’ and ‘how to find your voice’.	‘Digital body language—I do use this and I find it useful to provide information because you can actually see how they feel’.	‘NMC talks about the importance of confidentiality and using social media appropriately. I believe this would be a good tool to use to consolidate the importance of this for students and/or NHS staff’.	‘I will definitely use digital communication as most of the communication is done through emails, and it is very helpful’.	‘I think that in a setting where there is a lot of time spent on digital technology, it is important for people to understand it fully and how certain interactions can affect people in a detrimental way’.	‘Social media provides lots of information’. ‘It gives a good understanding of the importance of good digital communication’	‘I will use the core features of Microsoft Teams and how teams are brought together to create conversations and content’.	
Digital wellbeing	‘The resources in the digital wellbeing are useful and have good information. I also like the fact that there is a resource included for physical wellbeing for the neck as it is strained when using technology’.	‘I would use the resources provided, I already use certain features on my phone and laptop that stop me from using too much’.	‘I would use all resources provided in toolkit to help benefit digital wellbeing’.	‘I found the cyber security information useful and I would use it’. ‘I would use the digital wellbeing tool as this would help me in my personal and work life and how to keep a healthy balance between the two’	‘How to unwind and unplug—I feel that this is something that provides useful information’.	‘I would use the dealing with (digital) distraction as it provides helpful information’.	‘I didn't realise that digital wellbeing was not only covering cyberbullying etc. but also knowing when to take a break away from digital media’.	‘Will definitely use the neck stretching exercises & how to unplug and recharge’.	‘I liked the resources and felt they were relevant and addressed many topics within digital wellbeing’.	They would raise awareness on how to take time away from the screen and be aware of online dangers’.	‘It has made me think about it more and hopefully given me the opportunity and confidence to be more resilient around digital wellbeing and recognising cyber security’.	‘Spreads awareness of what the online world can be like’.
Digital research & information literacy	‘Reinforcing the practical skills of digital research, I agree we need more practical sessions on how to navigate digital research’.	‘I use Digital Research quite often in my practice and time at uni’.	‘I would use the videos to advance my use of excel’.	‘The information contained is relevant and can be a starting point for learning to do academic research and spotting the correct types of resources to utilise’.	‘RefWorks, Mentimeter both recommended’.	‘Using the internet as a source of data to conduct research, for example analysing posts online or articles’.	‘MANTRA and RefWorks looked very useful.’	‘I believe this competency is useful now in Uni whilst doing our degree so that we become proficient in finding relevant and reliable information. Mastering this competency now will also help whenever we graduate as it can be used to find info about a patient's condition and further reliable research’.	‘The information contained is relevant and can be a starting point for learning to do academic research and spotting the correct types of resources to utilise’.	‘I feel this is needed to help with essay work to ensure academic sources are valid’.	‘Use these when writing essays or finding evidence to back up the information I have written’.	
Digital creation	‘The information was clear and make it easier to understand what to do when creating different things’.	‘I would use them to make posters, power point presentations’.	‘Would use it to make presentations’.	‘How to create animated videos with PowerPoint and how to add voice in PowerPoint presentations’.	‘They look like they would be very helpful, and useful to learn about. Particularly animated videos with PowerPoint’	‘Can be useful when lecturers ask for assignments to be submitted in a certain format, that is, slide show for an audio submission’	“Getting your website online’. This provided useful information, and I would use this in the future if need be’.	‘I will definitely use digital communication as most of the communication is done through emails and it is very helpful’.				
ICT proficiency/ICT productivity	‘Explains what skills are required of students in order to use technology well’.	‘ICT proficiency competency is important as it provides information that covers the essential IT skills that are required in everyday life and needed to be successful in the workplace. Without coverage of these you many not complete tasks correctly’.	‘I would use the videos to advance my use of excel’.	‘MS Project management’.	‘This would help me learn more about how to take notes in a more creative way.’	‘It is a handy tool to use especially the ICT proficiency part for myself as my computer skills are not up to scratch and it is useful to have this tool to fall back on for support’.	‘I would use the videos to advance my use of excel’.					

### 5.3 Toolkit Evaluation Questions

#### 5.3.1 Quantitative Findings

Table 3 offers the main results related to the responses of students around the clarity of the toolkit content, its level of comprehension, and its content accessibility. Collectively, most of the students (87%, *n* = 294) agreed or strongly agreed that the content of the toolkit was clear. The results related to the comprehensiveness of the material were, similarly, positive, with most of the students indicating agreement or strong agreement with the statement ‘I found the content I reviewed at an appropriate level/easy‐to‐understand’ (88%, *n* = 298). The results related to the content accessibility also indicated positive experiences, with most of the students agreeing/strongly agreeing with the statement ‘I found the content easily accessible’ (87.5%, *n* = 292).

**TABLE 3:** Evaluation questions (clarity, comprehension, accessibility).

I found the content I reviewed clear	Number of responses	Percentage
Agree	215	63.6%
Strongly agree	79	23.4%
Neither agree nor disagree	29	8.6%
Strongly disagree	9	2.7%
Disagree	6	1.8%
**I found the content I reviewed at an appropriate level/easy‐to‐understand**	**Number of responses**	
Agree	214	63.30%
Strongly agree	84	24.90%
Neither agree nor disagree	25	7.40%
Strongly disagree	8	2.40%
Disagree	7	2.10%
**I found the content easily accessible**	**Number of responses**	
Agree	213	63.8%
Strongly agree	79	23.7%
Neither agree nor disagree	21	6.3%
Strongly disagree	16	4.8%
Disagree	5	1.50%

#### 5.3.2 Qualitative Findings

##### 5.3.2.1 Clarity of Content

Open comments were positive about the format of the toolkit, which was described as: ‘very easy to follow and didn't leave me feeling confused’, ‘it looks great as it is’, it's perfect’ and ‘it has all been very well done’, ‘really useful’, ‘full of information’, ‘very informative’, ‘have taken into consideration every aspect of issuing digital technologies’. Students found both the recommendations and the external links for various activities ‘very helpful and insightful’ as ‘lots of people aren't aware about the free productivity resources available’. The students also commented on how it targets health and wellbeing aspects associated with digital work and provides valuable foundational links to tools like SPSS and Excel, supporting users in building essential data literacy and analytical skills.

Other students highlighted the toolkit's ability to communicate efficiently and reach wider audiences, ‘in less time’ including both staff and students. They also praised its educational value, noting it introduced them to new and potentially useful platforms and resources ‘I have never heard of and could be useful’: ‘there's so many helpful resources that could be shared’.

##### 5.3.2.2 Level of Comprehension

Students provided positive comments on the quality of presentation, its clarity and layout as well as the ease of navigation. For example, they indicated that the toolkit is ‘detailed and informative’ with a ‘logical sequence’, it is ‘simple and comprehensive’, the content is ‘broken down well’ and it is ‘Easy to read and well put together’. Others explained that it has ‘clear and concise information’ and ‘a positive attitude’, is ‘informative’ and provides ‘clear definitions’, ‘a good pace’. Students found its style ‘very professional looking’, ‘Very advanced style! page‐turning’, commenting on the ‘use of links to videos’, the ‘bright and colourful’ style, ‘the use of ticks outlining what can be learned and the underlined/bold headings to break‐up the text’. Interestingly, one student also noted that ‘This is very useful as I often find it hard to get good sources of information that are relevant to me’, highlighting the value of synthesising useful information into one place, which other students also described as ‘signposting’: ‘…it gives the students a signpost on where they can go or what they can read’ and ‘I would use this as it signposts to useful information’.

##### 5.3.2.3 Content Accessibility

Students described the content as ‘Easy to read’ with ‘appropriate colours’ and ‘accessible font’. One student mentioned that ‘Lecturers can point students to the toolkit and it's an easy location for all students to access advice and resources’. Only two students raised concerns about the accessibility of the toolkit, particularly for non‐English speakers and learners with disabilities, despite acknowledging its informative content. The most significant feedback focused on improving usability for neurodivergent individuals, such as those with dyslexia. While the toolkit was seen as broadly useful, some found it challenging to stay engaged: ‘It's a bit difficult to stay involved from a neurodivergent perspective’. Suggestions included creating an alternative version featuring larger text, varied font colours, and read‐aloud options to better support diverse learning needs (Table 4).

**TABLE 4:** Issues encountered by neurodivergent students.

Audio	Text length/attention	Text size/font/background	Language complexity
‘I feel it would be easier to read if there was an audio button so the reader can follow along while it reads out the information’.	‘There is just way too much text and not enough space for it to be easily read. Please make a dyslexia friendly version’.	‘Great resource, might be good to make it inclusive meeting dyslexia friendly font styles and background etc. the writing is a bit small to read’	‘Some of the language used may be difficult for international students to understand. It could be simpler, or a glossary could be provided for people to understand…There is lots of good and informative information though’.
‘A read aloud version would be helpful too’.	‘It would be good to have the option to change the background colour as black on white is hard to read. Maybe larger print and less words please’.	‘None other than maybe use more colours to maintain engagement like different coloured headings so we can differentiate’.	‘I don't think it's very accessible for those whose English isn't their first language’.
‘Accessible for BSL users’.	‘The actual content in booklet is a bit too many words and overwhelming especially for somebody like me with dyslexia’.	‘Needs less words and maybe a bit more colour in text not just all black’.	
	‘I would use it, but as someone who struggles with attention to lots of writing on a screen I find this quite difficult’	‘Bigger text/less on each page’.	
	‘I haven't really understood the digital toolkit. I think this is mainly due to my processing issues’.	‘I think it needs to have bigger writing to make it easier to read’.	

### 5.4 Toolkit Implementation Ideas

#### 5.4.1 Quantitative Findings

Students were asked to select the most suitable stage in their course to implement the toolkit. Half of the respondents indicated that the toolkit should be implemented early in the course (50%, *n* = 168), followed by more than a third (39.3%, *n* = 132), who showed value for the toolkit in all the different stages of their course. One third of the students also expressed that the most suitable time would be before the course (33.3%, *n* = 112) (Table 5).

**TABLE 5:** Stage of utilising the toolkit.

Which stage of your studies do you consider the most ideal time for staff to utilise the toolkit to support developing your digital competencies?’	Number of responses	Percentage
Early in my course (e.g., in year one)	168	50%
All of the above	132	39.3%
Before starting my course (e.g., during induction)	112	33.3%
During the middle of my course	31	9.2%
At the end of my course (e.g., at the end of year three or four)	27	8%

#### 5.4.2 Qualitative Findings

Via open‐ended comments, students further explained their preference for implementing the toolkit early in their course. This could take the form of a ‘copy’ that could be ‘sent as an induction to new students’ and ‘it would be more beneficial before starting the course’, also pointing to a broader need to ‘Incorporate and promote these competencies in inductions to universities’. Students also felt that it would be in their first year ‘a good place to start’ and ‘for a general better understanding’ that would be ‘useful to people joining to see what skills are required of them or first years to learn’. Other students also suggested ‘Definitely introduce in Year 1 and progress throughout the three years’, ‘so people are aware of it for the whole course’ and ‘ready to use it in their studies’, offering ‘support’ to them ‘throughout university’. This emphasis on continuation and revisiting the toolkit as a reference point so that students could go back to it, if needed at different points within a course, was also reinforced by other students with initiative explanations such as: ‘Continuous utilisation of the toolkit, constant support for digital competencies through the course’ and ‘I would take time to go through the toolkit at different stages of my course’.

### 5.5 Relevance to Students' Learning/Practice and Career Development

#### 5.5.1 Quantitative Findings

With the focus of nursing courses on practical digital skills, it was important to find from students whether the toolkit would be relevant for strengthening practice‐based skills, particularly as they spent a considerable time of their course on clinical practice placements, which provided practical learning opportunities. When students were asked about the inclusion of the DCT for supporting practice placements, their responses were overall positive, with more than 80% of students selecting ‘agree’ and ‘strongly agree’, although almost one fifth of students were undecisive (19.6%). Beyond course‐related learning, it was also valuable to explore students' perceptions around the support provided by the toolkit for their future career development, expanding beyond study purposes. This showed that students endorsed the toolkit in this area, with more than half agreeing or strongly agreeing with the statement. Overall, it was clear that the toolkit had been received very positively by students, who found a strong potential for using it in their studies and for their future career trajectories. Beyond the personal and professional benefits mentioned above, most of the respondents (79.6%, *n* = 269) also indicated that they would recommend the toolkit to other students (Table 6).

**TABLE 6:** Learning, professional career development, and overall recommendation.

I would find the digital competencies toolkit relevant to support me in my practice placements	Number of responses	Percentage
Agree	206	61.1%
Neither agree nor disagree	66	19.6%
Strongly agree	46	13.6%
Disagree	13	3.9%
**I would find the digital competencies toolkit relevant to support me in my professional career development**	**Number of responses**	**Percentage**
Agree	217	44.2%
Strongly agree	58	17.2%
Neither agree nor disagree	47	13.9%
Disagree	10	3.0%
Strongly disagree	6	1.8%
**I would recommend the digital competencies toolkit to other students**	**Number of responses**	**Percentage**
Agree	199	58.9%
Strongly agree	70	20.7%
Neither agree nor disagree	54	16.0%
Disagree	9	2.7%
Strongly disagree	6	1.8%

#### 5.5.2 Qualitative Findings

Students' open comments also referred to practice‐based modules or practical activities, such as taking patient notes and time management. Interestingly, several students noted a wider need for students' development of digital skills related to the use of technology in general, with a suggestion to incorporate the toolkit into all the modules of the course or into all the courses of the university. Students made different suggestions for specific modules where the toolkit could be implemented, including those that focused on nursing‐specific tasks, such as health digital research, health promotion, simulated practice, and public health, but also those that addressed transferable and study skills, such as academic writing, presentations, and academic writing. One student recommended that it could be part of students' assessed coursework, although it may be difficult to find space for it, while a few students expressed interest in developing digital skills, but not as a priority: ‘…do not have lots of time to learn new IT skills’. Most students, however, articulated a positive attitude towards upskilling and dedicating time for that task: ‘Taking time to upskill yourself in digital skills is vital to continue to provide efficient work’. Students' qualitative comments further reinforced this finding, adding on how the toolkit would be useful for future career directions and practice, expanding beyond their own personal benefit and reflecting upon its usefulness for helping others, their everyday life, and thinking about their roles and practice overall: ‘It is important for us to be able to use our resources confidently, this workbook supplied us with posts and information that can help with that’. This demonstrated how they connected certain skills back to professional practice and values (Table 7).

**TABLE 7:** Implementing the toolkit in learning.

Not a priority	Priority	Useful for health‐related modules	Useful for study skills related modules	Useful to all modules/courses	Useful for future career directions	Wider implications
‘I would like to see it integrated within the coursework. It seems a beneficial tool but may be a lot of work to add on to an already busy course timetable’. ‘If needed I would make time to upskill myself but it wouldn't be a priority’. ‘We have so much to learn on the course but do not have lots of time to learn new IT skills and if mandatory as part of an assessment this will cause stress for many.’ ‘Some online sessions maybe on specific aspects if there is a university expectation to require the skill’.	‘I would easily donate some time to learn more about these things’. ‘I will dedicate time to upskill myself so I could get better’. ‘I would take time to upskill myself’. ‘I have dedicated time to upskill myself’. ‘I would take time to upskill myself to learn something new’. ‘I would take time to look at it myself and familiarise myself to it’. ‘Taking time to upskill yourself in digital skills is vital to continue to provide efficient work’.	‘Could relate to health promotion’. ‘I would possibly incorporate it in to simulated practices’. ‘Found it quite useful but would probably relate it to Public Health’. ‘I enjoyed the resources provided surrounding time management. Will be using these resources for Practice Learning Experience 2 module’. ‘I would maybe incorporate it into the Developing Nursing Practice module because it works best there. APLE (Alternative Placement Learning Environment) and ICT’. ‘Incorporate it in 1st year APLE (Alternative Placement Learning Environment) maybe?’ ‘Pre‐placement education would be useful’, ‘Just going over how to use technology to help in our placements would be good. It would probably have to be run through NHS but if there was a simulation for patient notes or something would be useful’.	‘This may be incorporated within the Research Methods modules’. Would incorporate into essay written modules in order to showcase my work’. ‘I think this would be useful to incorporate during academic writing modules’. ‘Incorporated into the course as an additional shorter module would be beneficial’. ‘It would have been good to have this resource for the last module which involved a presentation on PowerPoint which I had never created before’. ‘I think it would be useful to key introductory modules against it. Something we haven't had much of yet for example is data analysis and presentation, this is very important but hasn't been covered in any module yet that I am aware of. I would be very glad if we got the opportunity to take part in this’.	‘I think it would be useful for all modules’. ‘It looks like it would be relevant as a support for all our nursing modules’. ‘I think Uni should offer extra classes to improve your skills when it comes to technology’. ‘I think it would be good if it were included at the beginning of every course/degree at the university’. ‘Would be good to incorporate into most modules…the uni needs to teach more on digital literacy and IT information for practice learning environments’.	‘I found this very helpful as it displayed a ranged of different sources that could be used as a student nurse not only through RGU but also other networks such as LinkedIn. I will definitely be using this as I continue through my nursing career’. ‘I found this information useful as I believe that it is important for us as nurses in 2023, everything is moving online so it is important for us to be able to use our resources confidently, this workbook supplied us with posts and information that can help with that’. ‘Really enjoyed this course toolkit was very helpful and will be useful for the future’. ‘Very interesting reading, I would use this to keep with my course and career’ ‘I think I could use them during my course and when working after graduation’. ‘I would definitely use it in my nursing career’. ‘I would definitely use the resources and recommendations throughout my studies and future to support my learning, gain knowledge, and support other students and co‐workers’. ‘Technology in our environment is always changing so yes, definitely it is useful.’	‘…shows the importance of being able to use technology and how it impacts our practice. ‘It also goes in to how we can improve the wellbeing of society as well and how we can implement this’. ‘Would highlight the importance of digital literacy and how it links to health care provision’. ‘Everyday participation as digital citizenship skills will enable better performance academically, socially and at workplace’. ‘They provide lots of information that I find really useful and would take outside of studies and work’. ‘I think it would be useful as it can be used in everyday life so can apply to various situations’.

### 5.6 Suggestions for Further Toolkit Development

Students shared several suggestions on how the toolkit could be further developed in relation to reducing or adding to its content length. This involved ‘shortening the kit so it can be easily read and quickly understandable’, especially by non ‘tech‐savvy’ or by ‘people who struggle with digital technology’, although other students understood the need to incorporate explanations: ‘I am not the most computer savvy so it will help to have a basic knowledge like this booklet gives’ and ‘I do understand there will be a lot of information that will need to be explained’.

Interestingly, however, most of the other students' suggestions included creating additional content, such as preparing different tailored versions for diverse stages of a course with ‘different levels of toolkits we could work through as we progress in nursing’ as students in the later parts of a course would already know that ‘all our practice should be based on evidence and research’, or ‘why we collect research and findings and how to present them’. Other comments indicated a need for ‘more interactive content’ and offering ‘Refresher sessions on digital literacy’. This reinforced the idea that the length of the document would not be an issue, as selected parts could be more relevant for the different stages of a learning journey. Other students, however, noted that the inclusion of easy navigation tools, offering options for page skipping, and checklists navigation aids would be helpful: ‘Allowing you to skip to a page’, ‘Create button to say information out verbally in areas’, adding ‘a digital skills basics checklist’ and a ‘bullet point summary/glossary’. In addition, several suggestions were made by the students on adding further content and resources to different areas of the toolkit which could be considered in future versions of the toolkit (Table 8).

**TABLE 8:** Suggested areas for additional themes and design.

‘Digital proficiency’	‘Digital productivity’	‘Digital creativity’	‘Digital communication’	‘Digital identity management’	‘Digital wellbeing’
‘Uploading assessments in different formats’. ‘PowerPoint presentations with less MB *[megabytes]* so I can easily use it and send to others’.	‘Typing skills such as touch typing’. ‘Setting up everything on phone before you start’. ‘Spreadsheets how to set up and work’.	‘Presenting posters’ ‘Examples of how I can use creation of posters etc. in practice’.	‘Accessing, producing and understanding digital media’.	‘How to recognise cyber security breech and how to report these’. ‘Safe use due to hacking and fraud’.	‘Specific local wellbeing information, such as support groups and local mental health information’. ‘Points highlighted in the toolkit that may help our health and wellbeing e.g., reminder to take regular breaks, create a comfortable environment etc.’. ‘Focus on mental health resources or support instead of taking a ‘just switch off’ mindset’.

## 6 Discussion

### 6.1 Recommendations for Further Research

Further research to develop the toolkit should be ongoing as technological developments are advancing, and future versions of the toolkit should therefore incorporate more advanced ‘Healthcare 4.0’ skills, exploring ‘Big Data, the Internet of Things (IoT), and cloud computing’ (Gupta and Singh 2023) as well as artificial intelligence. However, these should be introduced with caution, catering for students’ individual differences and levels of digital skills when entering university to gradually address more advanced competencies in later stages of a course. Future research should also aim to incorporate the toolkit into students' learning over at least an academic semester to allow for deeper evaluation of its impact.

Additionally, by fostering a research‐oriented mindset, nursing schools can involve students in the continuing co‐planning/designing and co‐producing of the DCT that can create mutual value for both students and universities as students can act as co‐producers, change agents and partners in learning. That can help to enhance their educational experiences (Zarandi et al. 2022) and establish continuous feedback mechanisms, which are crucial for the success of digital skills development. Regular feedback from students can help to refine and improve the process and focus on developing content that is relevant and responsive to students' different needs as learners and as future professionals.

### 6.2 Implications for Policy and Practice

Based on the survey results, students indicated that the toolkit had the potential for positive impact in students' digital skills development for learning and practice and that it should be incorporated in different student learning trajectories. There was also a clear need for a design that supports different learning abilities and skill levels. As the values that underpin nursing are human centred with compassion and communication as fundamental values (Health Education England 2020), it was not surprising that students were particularly interested in two particular areas of the toolkit, ‘Digital Communication’ and ‘Digital Wellbeing’. These are closely aligned with nursing professional practice expected in ‘digital professionalism’, addressing both digital teamwork, such as that required for hybrid working practices, as well as efficient and compassionate communication with patients (Murray and Pérez 2014).

As noted earlier, there is also a need for continuous education in digital skills to meet technological changes and trends. For example, the areas of ‘ICT Proficiency’ in the toolkit could tackle more directly the use of health specific digital tools for e‐prescribing, e‐consultations and patient management. One possibility would be to make available training versions of the programs used in hospitals such as TrakCare Patient Management System (InterSystems 2024) or HEPMA (Hospital Electronic Prescribing and Medicines Administration) (NHS Scotland 2022) or other widely used programs to organise daily operations and reservations (e.g., Near Me 2024), especially when these are available as a training version for educational purposes. Technology also now expands to areas such as data analytics and artificial intelligence with a call for skills to evaluate the efficiency of data‐driven clinical decision‐making.

Future practice should also focus on embedding the DCT early into course assignments with the purpose of exploring the educational benefits of students' digital competencies development. Finally, students should be involved in the ongoing critical evaluation of their digital competencies and be committed to upskilling as a lifelong process. This will ensure that they can stay up to date with the latest technological advancements and continue to improve their digital skills throughout their careers.

### 6.3 Limitations of the Work

This study favoured inclusivity, empowered the student voice, and enabled student‐staff partnership. The direct involvement of students in the evaluation of the toolkit provided rich narratives and supported a student community that is valued via direct contribution to teaching and learning decision‐making processes (Akiva and Petrokubi 2016; Majee et al. 2022; Terry et al. 2019). The toolkit offered a holistic and discipline‐based approach, addressing several important digital skills. However, the study also presented two key limitations that should be considered in future work. Firstly, participants were asked to evaluate the toolkit without being able to immerse themselves in the actual development of digital skills, utilising the toolkit over a period of time. Secondly, the specific demographic characteristics of students who filled in the DCT evaluation were not available to shed further light on individual differences. Despite that, overall, the student‐focused, explorative nature of the study helped to evaluate the approach and allowed students to directly contribute to the future design of the toolkit. This was important as the DCT was designed as an organic document to be yearly updated to reflect students' changing needs, with new developments in digital skills.

## 7 Conclusions

The Ministerial led Nursing and Midwifery Taskforce of the Scottish Government has recently published a report highlighting a need for ‘strengthening digital literacy’ and increasing understanding of the opportunities available to nurses ‘to be involved in digital expansion, innovation and digital transformation with a plan to conduct a digital landscape mapping exercise (Scottish Government 2025). Providing essential training and resources to enhance digital skills at an early stage is therefore increasingly important for preparing a workforce that is well‐equipped to handle the technological demands of contemporary healthcare. It is important to foster a culture of digital literacy and innovation among nursing students, enabling them to be ready to leverage technology to improve patient care and streamline healthcare processes. This includes not only teaching students how to use healthcare technologies but also understanding emerging developments, such as those in data analytics and AI, and being able to apply digital tools in clinical practice safely and ethically. Incorporating digital literacy into nursing programs will ensure that graduates are well‐prepared for the demands of future digital healthcare. By fostering a research‐oriented mindset, nursing schools can help students develop the skills needed to drive innovation in healthcare.

This work employed co‐creation strategies in higher education that supported students to act as co‐producers, change agents and partners in learning in a way that enhances their educational experience (Zarandi et al. 2022). A co‐creation curriculum can lead to transformational experiences (e.g., positive relationships and community; engagement; taking risks, overcoming challenges and academic achievement and retention) (Lubicz‐Nawrocka and Bovill 2023). Students' insights and perspectives in this study demonstrated that incorporating co‐creation and participatory approaches in the design of digital literacy resources that aim to enhance the development of students' digital skills has the potential to engage students in ways that are relevant and effective for both learning and future professional practice. Students' evaluations were invaluable in understanding that the inclusion of the student voice and establishing continuous feedback mechanisms are crucial for the success of co‐creation activities that involve the development of digital skills. Regular feedback from students can therefore help to refine and improve the co‐creation process and focus on developing content that is relevant and responsive to students' different needs as learners and as future professionals. Keeping the toolkit up to date is also crucial; it needs to be evolving, adapting to technological advancements and digital behaviours constantly. However, this should not be a solo endeavour. Collaboration between institutions offering similar programmes and resources could lead to the development of more impactful and holistic digital competencies toolkit development projects, taking into consideration different socio‐cultural and contextual differences that play a role in students' learning.

## Author Contributions

K.M., E.S.L.: Made substantial contributions to conception and design, or acquisition of data, or analysis and interpretation of data; K.M., E.S.L.: Involved in drafting the manuscript or revising it critically for important intellectual content; K.M., E.S.L., M.P.H., K.N.K.: Given final approval of the version to be published. Each author should have participated sufficiently in the work to take public responsibility for appropriate portions of the content; K.M., E.S.L., M.P.H., K.N.K.: Agreed to be accountable for all aspects of the work in ensuring that questions related to the accuracy or integrity of any part of the work are appropriately investigated and resolved.

## Ethics Statement

This project was approved by the Research Ethics Committee of the School of Health at Robert Gordon University, under the Research Governance and Research Ethics Policies, with SERP reference number: 2334, dated 09/01/2023.

## Conflicts of Interest

The authors declare no conflicts of interest.

## Data Availability

The data that support the findings of this study are available from the corresponding author upon reasonable request.
